# Clinical outcome of fresh and vitrified-warmed blastocyst and cleavage-stage embryo transfers in ethnic Chinese ART patients

**DOI:** 10.1186/1757-2215-5-27

**Published:** 2012-10-05

**Authors:** Guo Qing Tong, Shan Ren Cao, Xun Wu, Jun Qiang Zhang, Ji Cui, Boon Chin Heng, Xiu Feng Ling

**Affiliations:** 1 Reproductive Medicine Center, Shuguang Hospital Affiliated to Shanghai University of Traditional Chinese Medicine, 528, Zhangheng Rd, Shanghai 201203, PR China; 2 Department of Reproduction, Nanjing Maternity and Child Health Hospital of Nanjing Medical University, Nanjing 210004, PR China; 3 ETH-Zurich, Department of Biosystem Science & Engineering, Mattenstrasse 26, Basel, 4058, Switzerland

**Keywords:** Blastocyst transfer, Vitrification, Clinical pregnancy, Implantation

## Abstract

**Objectives:**

This study sought to evaluate the outcome of fresh and vitrified-warmed cleavage-stage and blastocyst-stage embryo transfers in patients undergoing ART treatment within an ethnic Chinese population.

**Study design:**

We compared the clinical results of embryo transfer on the 3rd (cleavage stage) or 5th (blastocyst stage) day after oocyte retrieval, including clinical pregnancy rates, implantation rates and multiple pregnancy rates.

**Results:**

Our data showed that blastocyst transfer on day 5 did not significantly increase clinical pregnancy rate (41.07% vs 47.08%, p>0.05) and implantation rate (31.8% vs 31.2%, p>0.05) in patients under 35 years of age, in comparison with day 3 cleavage stage embryo transfer. In patients older than 35 years of age, the clinical pregnancy rate after blastocyst transfer was slightly decreased compared with cleavage stage embryo transfer (33.33% vs 42.31%, p>0.05). Unexpectedly, It was found that vitrified-warmed blastocyst transfer resulted in significantly higher clinical pregnancy rate (56.8%) and implantation rate (47%) compared with fresh blastocyst transfer in controlled stimulation cycles (41.07% and 31.8%, respectively). For patients under 35 years of age, the cumulative clinical pregnancy rate combining fresh and vitrified-warmed blastocyst transfer cycles were significantly higher compared to just cleavage-stage embryo transfer (70.1% versus 51.8%, p<0.05). However, the cumulative multiple pregnancy rates showed no significant difference between the two groups.

**Conclusions:**

In an ethnic Chinese patient population, fresh blastocyst transfer does not significantly increase clinical pregnancy rate. However, subsequent vitrified-warmed blastocyst transfer in a non-controlled ovarian hyperstimulation cycle dramatically improves clinical outcomes. Therefore, blastocyst culture in tandem with vitrified-warmed blastocyst transfer is recommended as a favourable and promising protocol in human ART treatment, particularly for ethnic Chinese patients.

## Introduction

In a typical ART treatment cycle, fresh cleavage-stage embryos are routinely transferred [1]. In recent years, with rapid progress in vitrification technology and blastocyst culture protocols, transfer of fresh and vitrified-warmed blastocysts, together with cryopreserved cleavage-stage embryos are now becoming more routine and commonplace in ART clinical practice [2,3]. Nevertheless, the optimal timing for embryo transfer has remained controversial to date.

A number of studies, as summarized in a Cochrane review [4], demonstrated the main advantages of blastocyst transfer, including better correlation between morphology and euploidy status and improved implantation potential due to better synchronization with the endometrium not adversely affected by controlled ovarian stimulation. These in turn translate to higher pregnancy and live birth rates after blastocyst transfer, as compared to cleavage-stage embryo transfer. Nevertheless, conflicting results were reported by a recent Cochrane meta-analysis, which found no evidence of any difference in pregnancy outcomes between Days 2-3 and 5-6 transfers of embryos [5]. Moreover, the same study [5] also found that blastocyst transfer was associated with an increase in failure to transfer any embryo in a cycle, as well as a decrease in embryo freezing rates. Interestingly, in a recent study by Langen et al. [6], it was reported that Asian ART patients (majority of Chinese ethnicity) had significantly poorer clinical outcome compared to Caucasians, even though there was no difference in blastocyst quality between the two populations. Hence, it is plausible to hypothesize that endometrial receptivity is more adversely affected by controlled ovarian stimulation in ethnic Chinese patients, as compared to Caucasian patients.

Embryo cryopreservation following ART cycles provided further possibilities of success, in addition to that achieved with fresh embryo transfer [7]. Therefore cumulative pregnancy rates after completion of both fresh and additional vitrified embryo transfers per oocyte retrieval cycle should be considered a more accurate measure of clinical outcome, rather than just pregnancy rate per embryo transfer cycle.

Our study retrospectively analyzed the clinical pregnancy, implantation and multiple pregnancy rates after transfer of fresh and vitrified-warmed blastocysts and cleavage-stage embryos in ethnic Chinese women undergoing ART treatment. The aim is to establish an optimized embryo transfer protocol in clinical ART practice, at least for ethnic Chinese patients.

## Materials and methods

### Patients

In order to minimize the influence of various subfertility factors, only patients younger than 40 years old were included in this study. From January 2009 to July 2010, a total of 551 couples including 478 patients younger than 35 years old and 73 patients older than 34 years old were evaluated retrospectively after the transfer of fresh or vitrified-warmed embryos at either the cleavage or blastocyst stage. The 478 and 73 patients were allocated to either cleavage-stage (n=310, <35years and n=52, ≥35, respectively) or blastocyst-stage (n=168 and 21, respectively) transfer. The primary outcomes being measured were the cumulative clinical pregnancy rates and multiple pregnancy rates.

### Ovarian stimulation

Standard long protocol for ovarian stimulation was used for all ART patients, as described previously [8]. Briefly, this involved down-regulation with GnRH agonist triptolerin (0.1mg S.C. daily until FSH administration, then reduced to half dose daily) (Decapeptyl, lpsen-biotech, Paris, France) followed by daily injections with recombinant FSH (Serono, Switzerland). Follicular growth was monitored with ultrasound and measurement of blood levels of oestradiol, progesterone and LH concentrations. Urinary human chorionic gonadotrophin (HCG: Lizhu, Zhuhai, China) was administered at a dosage of 5000-10000 IU(intramuscular) when there were more than two follicles measuring 18 mm in diameter. Luteal-phase support with intramuscular injections of progesterone (60mg daily) was administered on the day of oocyte retrieval. Transvaginal and ultrasound-guided follicular aspiration was performed 34-36 h after HCG injection. The choice of either IVF or ICSI procedure was determined by semen analysis parameters. IVF and ICSI procedures were performed as previously described [8].

### Blastocyst culture system

Oocyte cumulus complexes were identified, washed in culture medium (G-IVF; Vitrolife, Gothenburg, Sweden) under inverted microscope and were cultured in groups of four or five with 0.9 ml of culture medium per well inside the tri-gas incubator (Personal MultiGas CO_2_ incubator, APM-30D, ASTEC, Japan) at 37°C in an environment of 6% CO_2_, 5% O_2_ and 89% N_2_. The washed oocytes were then inseminated with either 50,000—100,000 normal motile spermatozoa (5 hours incubation) or by ICSI after denudation 2-4 h after pick-up. Subsequently, the inseminated oocytes were transferred into 50***μ***l drops of fresh pre-equilibrated culture medium (G1.5; Vitrolife) under 3.5 ml of sterile paraffin oil (Vitrolife). The following day, the oocytes were checked for fertilization status and then cultured in groups of two to three in fresh medium under oil for a further 2 days (2- or 8-cell stages). Scoring of day-3 embryos were performed according to previously published criteria including cell number, regularity of the blastomeres, fragmentation and morphological aspects such as granulation [8]. Day-3 embryos were thus scored on a scale of 1 (high grade) to 4 (low grade). In the blastocyst transfer group, the embryos were then regrouped according to their similarity of cell stage and embryo score and were cultured in groups of two to three in fresh medium under oil from days 3 to 5. Subsequently, the embryos were then transferred to fresh G2.5 medium and cultured for one more day. The blastocysts were scored according to Gardner’s standard on day 5 and day 6 as described previously [9]. In the cleavage-stage embryo transfer group, only top-quality embryos (6- or 8- cell stage with less than 20% fragmentation, and with symmetrical or very slightly asymmetrical blastomeres) [8] were either transferred or vitrified on Day3. Culture of the other non-top-quality embryos was extended for further 2-3 days until blastocysts were formed. All surplus embryos of good quality on day 3, day 5 or day 6 were cryopreserved through vitrification.

### Protocol for vitrification and warming

The cleavage-stage embryos and expanded blastocysts were vitrified and warmed according to the method developed by Kuwayama et al [10,11]. Embryos were vitrified by using the commercially-available Cryotop device and vitrification solutions (Kitazato BioPharma Co., Japan). The first equilibration was performed in 7.5% ethylene glycol (EG) and 7.5% dimethylsulfoxide(DMSO) at room temperature for 8-10min. Subsequently, embryos were transferred to 15% EG, 15% DMSO and 0.5 M sucrose for 1 min, and then placed on the film strip of the Cryotop within a single small drop. The excess solution was removed to leave just a thin layer around each embryo and the Cryotop was submerged into liquid nitrogen, with the strip being covered with a cap and the sample was stored submerged in liquid nitrogen. Upon warming, the cap was removed under liquid nitrogen and the film strip of Cryotop was quickly submerged for 1 min in 1 ml of 37°C warming solution containing 1.0 M sucrose, followed by transfer of the embryos to a room temperature solution containing 0.5 M sucrose, and further incubation for 3 min. After two subsequent wash procedures in basic medium at room temperature for 10 min in total, the embryos were transferred into 50***μ***l of culture medium (G2.5; Vitrolife).

### Endometrium preparation

A crucial factor for implantation in vitrified-warmed embryo transfer is exact synchronization between endometrial maturation and embryo development [12]. Vitrified-warmed embryo transfer has been successfully performed during a natural cycle after spontaneous ovulation [13] or after artificial preparation of the endometrium with exogenous steroids [14]. During artificial cycles, Estradiol Valerate (Schering, Zydus, Germany) was administered orally at 3mg twice daily, from Day 2 to Day 7 of the menstrual cycle. After ultrasonography confirmed an endometrial thickness exceeding 10 mm, Estradiol Valerate was administered for another 3 more days and the administration of progesterone (60mg i.m. daily) was initiated. If the endometrial thickness was <7 mm, Estradiol Valerate dose was increased from 4 mg to 6 mg twice daily in several cases.

### Embryo transfer

Patients who have four or more than four top grade embryos on day 3 (6- or 8- cell stage with less than 20% fragmentation, and with symmetrical or very slightly asymmetrical blastomeres) [8] , were selected for blastocyst transfer. Those patients with less than four top grade embryos on day 3 had cleavage-stage embryo transfer on day 3. Embryo warming was scheduled on day 3 or day 5 afternoon after the initiation of progesterone administration, with vitrified-warmed embryo transfer being performed on the next day. The time duration from warming to transfer ranged from 14-16h. Embryo transfer (a maximum of three embryos per transfer) was performed under ultrasound guidance. Luteal-phase support was achieved with intramuscular injections of 60 mg of progesterone daily for two weeks. Serum hCG concentrations were measured 14 days after embryo transfer, and clinical pregnancy rates per embryo transfer cycle and implantation rates per transferred embryos were based on the detection of fetal heart beats by ultrasound, after 5 weeks following embryo transfer.

### Statistical analysis

The results were analyzed statistically by either the Student’s *t*-test for comparison of mean values or the chi-squared test for comparison of percentages using Statistical Package for Social Science version 13.0 (SPSS,USA). A value of P<0.05 was considered to be statistically significant.

## Results

A total of 478 couples under 35 years of age were assigned to either cleavage-stage embryo transfer (n =310 patients) or blastocyst transfer (n =168 patients). There were no significant differences between the two groups with respect to patient clinical characteristics except for age (Table 1). At the same time, a few patients more than 34 years of age were also assigned to undergo embryo transfer of either cleavage-stage embryos (n=52 patients) or blastocysts (n=21 patients). Again, patient clinical characteristics were not statistically different between the two groups for patients above 34 years of age (Table 2).

**Table 1:** Patient characteristics for blastocyst and cleavage-stage transfer: Jan 2009 ~ Jul 2010 (age < 35 years)

	**Cleavage-stage transfer group**	**Blastocyst transfer group**
Number of patients	310	168
Female patient age (y, mean± SD)	29.61±3.03	28.87±2.70^a^
Duration of infertility (y, mean± SD)	3.90±1.88	3.92±1.90
Body mass index (w, mean± SD)	21.88±2.54	21.84±2.24
ICSI cycle, no. of patients (%)	90( 29.0)	45(26.8)
Tubal factor, no. of patients (%)	151( 48.7)	83(49.4)
Male factor, no. of patients (%)	43(13.9 )	23(13.7)
Tubal and male factor, no. of patients (%)	89( 28.7)	52(31.0)
Multiple factors, no. of patients (%)	27( 8.7)	10(6.0)

Note: Values are presented as number, number (%) or mean ± SD.

ICSI: intracytoplasmic sperm injection.

Duration of infertility, body mass index, percentage of ICSI cycles and percentage of different infertility factor were all not significantly different between cleavage-stage and blastocyst transfer group.

a:significant difference between the cleavage-stage and blastocyst transfer groups (P<0.05) .

**Table 2:** Patient characteristics for blastocyst and cleavage-stage transfer: Jan 2009 ~ Jul 2010 (age between 35 to 39 years)

	**Cleavage-stage transfer group**	**Blastocyst transfer group**
Number of patients	52	21
Female patient age (y, mean± SD)	36.69±1.45	37.05±1.32
Duration of infertility (y, mean± SD)	5.85±3.31	6.45±3.20
Body mass index (w, mean± SD)	22.14±2.07	22.84±2.47
ICSI cycles, no. of patients (%)	12(23.1)	5(23.8)
Tubal factor, no. of patients (%)	33(63.5)	10(47.6)
Male factor, no. of patients (%)	4(7.7)	4(19.0)
Tubal and male factor, no of patients (%)	13(25)	5(23.8)
Multiple factors, no. of patients (%)	2(3.8)	2(9.5)

Note: Values are presented as number, number (%) or mean ± SD.

ICSI: intracytoplasmic sperm injection.

Female patient age, duration of infertility, body mass index, percentage of ICSI cycles and percentage of different infertility factors were all not significantly different between the cleavage-stage and blastocyst transfer groups.

In fresh embryo transfer cycles, the number of retrieved oocytes in the blastocyst transfer group was significantly higher than the cleavage-stage embryo transfer group (Table 3, younger than 35 years: 11.43±4.01 versus 7.33±3.57, P<0.05; older than 34 years: 11.24±4.55 versus 6.23±3.68, P<0.05). Additionally, the number of embryos transferred was significantly different between the two groups (Table 3) among patients less than 35 years old (1.75±0.34 versus 2.15±0.55, P<0.05), as well as among patients aged older than 34 years old (1.76±0.83 versus 2.67±0.76, P<0.05).

**Table 3:** Clinical outcome after fresh blastocyst and cleavage-stage transfer

	**Female age < 35 years**		**35 years ≤Female age≤39 years**	
	**Cleavage-stage transfer group**	**Blastocyst transfer group**	**Cleavage-stage transfer group**	**Blastocyst transfer group**
No. of patients	274	168	52	21
Retrieved oocytes (mean± SD)	7.33±3.57	11.43±4.01^a^	6.23±3.68	11.24±4.55^a^
No. of embryos transferred (mean± SD)	2.15±0.55	1.75±0.34^a^	2.67±0.76	1.76±0.83^a^
Clinical pregnancy rate, no. (%)	129/274(47.1)	69/168(41.1)	22/52(42.3)	7/21(33.3)
Implantation rate, no. (%)	184/589(31.2)	97/305(31.8)	30/139(21.6)	8/37(21.6)
Multiple pregnancy rate, no. (%)	53/129(41.1)	28/69(40.6)	7/22(31.8)	1/7(14.3)
Singleton pregnancy rate, no.	76	41	15	6
Twin pregnancy rate, no.	51	27	6	1
Triplets pregnancy rate, no.	2	1	1	0

Note : Values are presented as number, number (%) or mean ± SD.

a:significant difference between cleavage stage and blastocyst transfer in two different age groups (p<0.05).

Clinical pregnancy, implantation, and multiple pregnancy rates all show no significant difference between cleavage stage and blastocyst transfer in two different age groups.

For patients under 35 years of age, the clinical pregnancy rates (Table 3) in the cleavage-stage embryo transfer group versus blastocyst transfer group were 47.1% versus 41.7% ( P>0.05) respectively; while the corresponding implantation rates (Table 3) were 31.2% versus 31.8% ( P>0.05) respectively. For patients more than 34 years of age, the clinical pregnancy and implantation rates after cleavage-stage embryo transfer and blastocyst transfer (Table 3) also exhibited no significant differences (42.3% versus 32.3%, P>0.05; 21.6% versus 21.6%, P>0.05, respectively). The rate of multiple births after cleavage-stage embryo transfer and blastocyst transfer were 41.1% versus 40.6% (< 35 years old), and 31.8% versus 14.3% (> 34 years) respectively, and these were not significantly different for both groups of patients (Table 3).

The clinical outcomes of the blastocyst and cleavage-stage embryo transfer groups (under 35 years of age) with respect to fresh and vitrified-warmed transfer cycles are shown in Table 4. This data set includes 36 of 310 patients who did not undergo fresh cleavage-stage embryo transfer due to poor endometrial receptivity on Day3 (and consequently had all embryos vitrified at the cleavage stage). Out of 168 patients undergoing fresh blastocyst transfer in Table 4, 69 patients became pregnant (41.7%), leaving behind 99 unsuccessful patients. Among these 99 patients, 7 patients did not possess any supernumerary blastocyst for vitrification. Therefore, only 92 patients had vitrified-warmed blastocyst transfer. At the same time, out of a total of 310 patients who had fresh and vitrified-warmed cleavage-stage embryo transfer in Table 4, there were 26 patients who after having failed to get pregnant upon day 3 transfer, still possessed supernumerary embryos that formed blastocysts on day 5. These were subsequently vitrified and transferred in another cycle. Hence, there was a total number of 92 plus 26 = 118 patients having vitrified-warmed blastocyst transfer in Table 4.

**Table 4:** Clinical outcome after vitrified blastocyst and cleavage-stage transfer (age <35 years)

	**Cleavage-stage transfer**		**Blastocyst Transfer**	
	**Fresh group**	**Vitrified group**^**C**^	**Fresh group**	**Vitrified group**
No. of patients	274	36	168	118
No. of embryos transferred (mean± SD)	2.15±0.55	2.19±0.40	1.82±0.38	1.69±0.58^a^
Clinical pregnancy rate, no. (%)	129/274 (47.8)	18/36 (50.0)	69/168 (41.7)	67/118 (56.8)^b^
Implantation rate, no. (%)	184/589 (31.2)	27/79 (34.2)	97/305 (31.8)	94/200 (47.0)^b^
Mutiple pregnancy rate, no. (%)	53/129 (41.1)	9/18 (50.0)	28/69 (40.6)	26/67 (37.3)
Singleton pregnancy rate, no.	76	9	41	41
Twin pregnancy rate, no.	51	9	27	25
Triplets pregnancy rate, no.	2	0	1	1

Note:values are presented as number, number (%) or mean ± SD.

a:significant difference between cleavage-stage transfer and blastocyst transfer in vitrified group (p<0.01).

b:significant difference between fresh group and vitrified group in blastocyst transfer (p<0.05).

C: patients without transfer in fresh cycles.

Although different criteria were used in allocating patients to the vitrified blastocyst and vitrified cleavage-stage embryo transfer groups, the two data sets in Table 4 are still comparable, because both cases involved non-controlled ovarian hyperstimulation (non-COH) cycles. Additionally, it can be presumed that the 118 patients in the vitrified blastocyst transfer group were previously unsuccessful due to sub-optimal endometrial receptivity in their COH cycle, which would mirror the situation in the vitrified cleavage-stage embryo transfer group.

As seen in Table 4, neither the clinical pregnancy rate nor implantation rate differed significantly for cleavage-stage embryo transfer in the fresh versus vitrified-warmed group (47.8% versus 50.0%; 31.2% versus 34.2% respectively). In contrast, both clinical pregnancy and implantation rates were significantly different between fresh versus vitrified-warmed blastocyst transfer cycles (41.7% versus 56.8% and 31.8% versus 47.0%, respectively, P<0.05). Multiple pregnancy rates were similar in both groups among either cleavage-stage embryo transfer or blastocyst transfer cycles (Table 4).

The cumulative clinical pregnancy rates for fresh and vitrified-warmed embryo transfers are summarized in Table 5. Out of 310 patients who had day 3 cleavage-stage embryo transfer (Table 4), 26 unsuccessful patients had remaining surplus embryos that were cultured up to the blastocyst stage and vitrified, prior to being transferred in a subsequent vitrified-warmed cycle. This would therefore leave behind a total of 310 – 26 = 284 patients in the cleavage-stage embryo transfer group of Table 5. At the same time, 26 patients were added to 168 patients (Table 4) to give a cumulative total of 194 patients in the blastocyst transfer group of Table 5. In the blastocyst transfer group, 67 additional clinical pregnancies were achieved following vitrified embryo transfers, whereas only 18 additional clinical pregnancies were achieved in the cleavage-stage embryo transfer group (Table 5). Hence, the cumulative clinical pregnancy rate was significantly higher in patients aged less than 35 years after blastocyst transfer, as compared to patients with cleavage-stage embryo transfers (70.1% versus 51.8%, p<0.05). There was however no significant difference in the cumulative multiple pregnancy rates of both groups.

**Table 5:** Cumulative clinical outcome combining fresh and vitrified-warmed cycles in blastocyst and cleavage-stage transfer (age < 35 years)

	**Cleavage-stage transfer**	**Blastocyst Transfer**
No. of couples	284	194
Overall clinical pregnancies per couple, no. (%)	147/284(51.8)	136/194(70.1)^a^
Fresh embryo transfer, no.	129	69
Vitrified-warmed embryo transfer, no.	18	67
Cumulative multiple pregnancy rate, n (%)	62/147(42.2)	54/136(39.7)
Singleton pregnancy rate, no.	85	82
Twin pregnancy rate, no.	60	52
Triplets pregnancy rate, no.	2	2

Note : Values are presented as number or number (%).

a:significant difference between cleavage-stage transfer and blastocyst transfer groups (p<0.05).

## Discussion

The ultimate aim of an ART procedure is to achieve one singleton live birth per stimulated cycle. However, it is still unclear which embryo transfer model will achieve optimal results for ART patients. To try to resolve this question, 551 couples were assigned to fresh or vitrified-warmed embryo transfer at either the cleavage stage or blastocyst stage for their first or second ART attempt.

In accordance with local ART regulations, and due to an anticipated enhancement of implantation rate with blastocyst transfer, we limited the number of embryos transferred per cycle to three or less. Therefore, a maximum of three embryos were placed in each cryotop during vitrification. This is reflected in significantly lesser number of embryos being transferred in the blastocyst transfer group compared to the cleavage-stage transfer group. Hence, a trend towards lower multiple pregnancy rates was therefore observed in blastocyst transfer cycles. Nonetheless, there was no significant difference between the two groups with respect to implantation rate. Meanwhile our retrospective meta-analysis showed that the transfer of fresh blastocysts on day5 did not significantly increase clinical pregnancy rate and implantation rate within the general ART patient population compared with day3 cleavage-stage embryo transfer. In fact, the rates for blastocyst transfer were slightly decreased compared with cleavage-stage transfer, particularly in patients aged older than 34 years.

These findings are consistent with another recent study [15], which also reported that blastocyst culture and transfer reduced implantation and pregnancy rates in the general ART patient population compared to cleavage-stage embryo transfer. Nevertheless, positive results of blastocyst transfer have been reported by many previous studies, which all revealed higher implantation and pregnancy rates with blastocyst transfer compared to cleavage-stage embryo transfer [16-20]. It is difficult to explain why the results of our study demonstrated negligible advantages of blastocyst transfer, despite comparable baseline clinical characteristics, other than the patient age being significantly younger and the number of oocytes retrieved being significantly higher in the blastocyst transfer group. Nevertheless, while all of the above differences should be considered advantageous, being good prognostic factors for both blastocyst formation and pregnancy [21], this potential advantage did not translate into higher implantation and pregnancy rates for the blastocyst transfer group in this study. Several studies have underlined the difficulties of correctly selecting the best embryo on Day 2-3 [22,23]. The aim of extending embryo culture to Day 5/6 was to select an embryo with increased probability of implantation rather than just to improve embryo quality [19]. Another recent meta-analysis [24] suggested that the putative clinical superiority of blastocyst transfer protocols needs to be further verified and that the cumulative clinical pregnancy rate combining both fresh and cryopreserved embryo transfer cycles would be the more reliable measurable outcome for comparison.

In this study, it was surprisingly observed that vitrified-warmed blastocyst transfer for patients aged less than 35 years resulted in significantly higher clinical pregnancy and implantation rates compared with fresh blastocyst transfer. Our results are consistent with a previous study that reported significantly higher ongoing pregnancy, clinical pregnancy, and implantation rates in cryopreserved versus fresh embryo transfer cycles [25]. Differences in implantation rates between the two groups may reflect different endometrial receptivity and a higher degree of synchronization between endometrial development and the transferred blastocysts in vitrified-warmed cycles [26]. Our overall results demonstrated that in women undergoing their first or second ART attempt with vitrified-warmed embryo transfers, the cumulative clinical pregnancy rate was significantly increased upon blastocyst transfer, as compared to the transfer of cleavage-stage embryos. It is suggested that if embryo-endometrium synchrony was suboptimal, the embryos should be cryopreserved for a subsequent transfer under more optimal conditions [27]. Therefore, the concept of cryopreserving all available embryos and transferring them in subsequent non-stimulated cycles may enhance embryo-endometrium synchrony and clinical outcomes of ART cycles in some patients. This in turn may provide a number of clinical benefits, including increasing the cumulative pregnancy rates, reducing the risk of OHSS and decreasing patient discomfort and cost without the need for superovulation. Although a trend towards lower cumulative multiple pregnancy rate was also noticed in blastocyst transfer cycles, this still needs to be further improved and diminished to a minimum by modifying the embryo transfer strategy.

The results of this study therefore demonstrate that blastocyst culture and transfer is not suited for all ART patients. This could be because later embryonic development to the blastocyst stage and beyond is also dependent on maternally transcribed mRNA stored within the oocyte [28]; and we hypothesize that some patient with poorer quality oocytes (i.e. older women) may have abnormally low levels of such stored mRNA transcripts, leading to arrest of blastocyst formation. Examples of maternally transcribed mRNA that play important roles in blastocyst formation and beyond include CDX2 involved in trophectoderm function and maintenance of the blastocoelic cavity [29], and STK40 implicated in extra-embryonic endoderm differentiation [30].

Hence, blastocyst culture and transfer should be offered primarily to younger patients (less than 35 years) with better prognosis, in tandem with blastocyst vitrification. Undoubtedly, blastocyst transfer will still remain a favourable and promising option in ART. In future, the efficacy of embryo transfer at the blastocyst stage could be fully optimized through further refinement and improvement of current blastocyst cryopreservation protocols.

In any case, the ultimate aim of reproductive medicine practitioners would still be the improvement of ART efficacy in terms of take-home-baby rates. However, the optimal outcome in current ART practice is the delivery of singleton infants rather than multiple births. To this end, blastocyst culture in tandem with blastocyst vitrification and single-blastocyst transfer emerges as a highly efficient protocol in human ART treatment.

## Competing interest

All co-authors hereby declare that they have no competing interests in the production of this manuscript.

## Authors’ contribution

G.Q.T and X.F.L contributed to data collection and analysis, as well as manuscript writing. S.R.C, X.W, J.Q.Z and J.C contributed to data collection and analysis. B.C.H contributed to manuscript writing.
